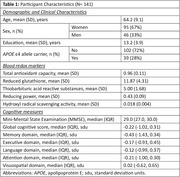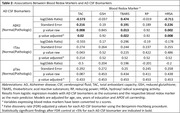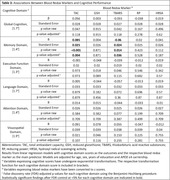# Association of Blood Redox Markers with Alzheimer Disease Biomarkers and Cognitive Performance

**DOI:** 10.1002/alz.085708

**Published:** 2025-01-09

**Authors:** Sokratis Charisis, Eva Ntanasi, Eirini Mamalaki, Zoi Skaperda, Adrian Noriega de la Colina, Demetrios Kouretas, Nikolaos Scarmeas

**Affiliations:** ^1^ Glenn Biggs Institute for Alzheimer’s & Neurodegenerative Diseases, University of Texas Health Sciences Center at San Antonio, San Antonio, TX USA; ^2^ Aiginition Hospital, National and Kapodistrian University of Athens, Medical School, Athens Greece; ^3^ Harokopio University, Athens Greece; ^4^ University of Thessaly, Volos, Thessaly Greece; ^5^ Department of Neurology and Neurosurgery, McGill University, Montreal, QC Canada; ^6^ Taub Institute for Research in Alzheimer's Disease and the Aging Brain, The Gertrude H. Sergievsky Center, Columbia University Medical Center, New York, NY USA; ^7^ Aiginition Hospital, National and Kapodistrian University of Athens Medical School, Athens Greece

## Abstract

**Background:**

Oxidative stress has been implicated in the pathogenesis of Alzheimer’s disease (AD). Nevertheless, whether redox perturbations are associated with cognition and AD pathology in the preclinical AD stages, remains unclear. We examined associations of blood redox markers with AD biomarkers and cognitive performance in older adults without clinical dementia.

**Method:**

In a sample of 141 participants without clinical dementia from a memory clinic‐based cohort, we measured total antioxidant capacity (TAC), reduced glutathione (GSH), thiobarbituric acid reactive substances (TBARS), reducing power (RP), and hydroxyl radical scavenging activity (HRSA), using standard laboratory techniques and absorbance spectrophotometry (Hitachi U‐1500). Cerebrospinal fluid (CSF) AD biomarkers, including Aβ42, pTau, and tTau, were measured using automated assays (Elecsys, Roche Diagnostics). Global and domain‐specific (i.e., memory, executive function, language, attention, and visuospatial abilities) cognitive performance was assessed with a comprehensive neuropsychological test battery. Associations of blood redox markers with AD biomarkers and cognitive performance were examined with logistic and linear regression models, respectively. Models were adjusted for age, sex, education, and APOE ε4 carriership. The false discovery rate for each outcome was controlled at <5% using the Benjamini‐Hochberg procedure.

**Result:**

Mean (SD) age was 64.2 (9.1) years and 95 (67%) of the participants were women (Table 1). Higher TAC and HRSA values were associated with lower odds for pathologically low CSF Aβ42, whereas higher TBARS values were associated with higher odds for pathologically low CSF Aβ42 (Table 2). Higher TAC values were associated with better memory cognitive domain scores, whereas higher TBARS values were associated with worse memory cognitive domain scores (Table 3).

**Conclusion:**

Higher blood TAC and HRSA, indicating better blood reactive oxygen species‐buffering capacity, were associated with lower odds for pathological CSF Aβ42, whereas higher TBARS, indicating increased lipid peroxidation, was associated with higher odds for pathological CSF Aβ42. Overall, these findings strongly point towards a link between redox imbalance and subclinical AD‐related neuropathology, and highlight the potential role of blood redox markers in AD risk stratification.